# Secretome of apoptotic peripheral blood cells (APOSEC) attenuates microvascular obstruction in a porcine closed chest reperfused acute myocardial infarction model: role of platelet aggregation and vasodilation

**DOI:** 10.1007/s00395-012-0292-2

**Published:** 2012-08-17

**Authors:** K. Hoetzenecker, A. Assinger, M. Lichtenauer, M. Mildner, T. Schweiger, P. Starlinger, A. Jakab, E. Berényi, N. Pavo, M. Zimmermann, C. Gabriel, C. Plass, M. Gyöngyösi, I. Volf, H. J. Ankersmit

**Affiliations:** 1 Department of Thoracic Surgery, Medical University of Vienna, Vienna, Austria; 2 Christian Doppler Laboratory for Cardiac and Thoracic Diagnosis and Regeneration, Vienna, Austria; 3 Institute of Physiology, Medical University of Vienna, Vienna, Austria; 4 Department of Dermatology, Medical University of Vienna, Vienna, Austria; 5 Department of Surgery, Medical University of Vienna, Vienna, Austria; 6 Department of Biomedical Laboratory and Imaging Science, University of Debrecen, Debrecen, Hungary; 8 Red Cross Transfusion Service for Upper Austria, Linz, Austria; 9 Department of Cardiology, Medical University of Vienna, Vienna, Austria

**Keywords:** Microvascular obstruction, Acute myocardial infarction, Platelet function, Vasodilation, No-reflow, PBMC, Paracrine factors

## Abstract

Although epicardial blood flow can be restored by an early intervention in most cases, a lack of adequate reperfusion at the microvascular level is often a limiting prognostic factor of acute myocardial infarction (AMI). Our group has recently found that paracrine factors secreted from apoptotic peripheral blood mononuclear cells (APOSEC) attenuate the extent of myocardial injury. The aim of this study was to determine the influence of APOSEC on microvascular obstruction (MVO) in a porcine AMI model. A single dose of APOSEC was intravenously injected in a closed chest reperfused infarction model. MVO was determined by magnetic resonance imaging and cardiac catheterization. Role of platelet function and vasodilation were monitored by means of ELISA, flow cytometry, aggregometry, western blot and myographic experiments in vitro and in vivo. Treatment of AMI with APOSEC resulted in a significant reduction of MVO. Platelet activation markers were reduced in plasma samples obtained during AMI, suggesting an anti-aggregatory capacity of APOSEC. This finding was confirmed by in vitro tests showing that activation and aggregation of both porcine and human platelets were significantly impaired by co-incubation with APOSEC, paralleled by vasodilator-stimulated phosphoprotein (VASP)-mediated inhibition of platelets. In addition, APOSEC evidenced a significant vasodilatory capacity on coronary arteries via p-eNOS and iNOS activation. Our data give first evidence that APOSEC reduces the extent of MVO during AMI, and suggest that modulation of platelet activation and vasodilation in the initial phase after myocardial infarction contributes to the improved long-term outcome in APOSEC treated animals.

**Electronic supplementary material:**

The online version of this article (doi:10.1007/s00395-012-0292-2) contains supplementary material, which is available to authorized users.

## Introduction

Myocardial infarction remains one of the major health issues worldwide. Early reperfusion of the culprit coronary artery within a narrow time window by percutaneous coronary intervention (PCI) and fibrinolytic agents has significantly improved early mortality [59]. Although tremendous efforts have been made in replacing infarcted myocardium, so far no therapy has proven effective in clinical application. The induction of myocardial repair by progenitor cells was suggested a promising strategy based on encouraging data from animal models [15, 23, 47, 48, 63]. However, the efficacy of stem cells as therapeutic agents in human AMI is currently under scrutiny [22, 26, 40, 62]. Based on recent observations showing that the infusion of cultured apoptotic peripheral blood mononuclear cells (PBMC) was able to prevent experimental AMI in rodents [4, 38] we speculated whether paracrine factors secreted from PBMC—termed APOSEC (abbreviation for APOptotic cell SECretoma)—are capable to attenuate AMI in a rodent and in a closed chest porcine ischemia/reperfusion AMI model. By a single intravenous infusion of APOSEC, scar tissue formation was significantly reduced. Additionally, an improvement of haemodynamics with higher values of ejection, and a better cardiac output was found in magnetic resonance imaging (MRI) analyses. A possible mode of action was suggested by showing that co-incubation of primary human cardiomyocytes with APOSEC led to an activation of pro-survival signalling-cascades (AKT, Erk1/2, CREB, c-Jun), and increased anti-apoptotic gene products (Bcl-2, BAG-1) in vitro, consequently protecting cardiomyocytes from starvation-induced cell death [39].

However, this ascribed mechanism only partially explains the beneficial effects of the “biological” APOSEC in AMI. Although coronary blood flow is re-established after PCI, no-reflow phenomena impair the beneficial effect of reperfusion due to microvascular obstruction (MVO) [51]. The two major pathophysiological mechanisms associated with MVO are enhanced platelet activation in the microcirculation and coronary vasoconstriction. Clinical reports have evidenced that platelet activation is directly correlated with the severity of myocardial damage after AMI [8, 16, 17]. Besides, there is sufficient evidence that the vasomotor state in the coronary vasculature is closely linked to the no-reflow phenomenon [50, 60]. Based on these accepted pathophysiological concepts we speculated whether APOSEC treatment has an effect on the development of hypoxia-induced MVO.

Here, we provide evidence that intravenous application of APOSEC attenuates myocardial infarction by reducing MVO in a porcine closed chest ischemia/reperfusion AMI model. Moreover, we show that APOSEC is an anti-aggregatory compound and has vasodilatory properties.

## Materials and methods

### Generation of porcine and human APOSEC

For large animal experiments, blood was obtained from pigs by direct heart puncture under sterile conditions. Peripheral blood mononuclear cells were purified by Ficoll-Paque (GE Healthcare Bio-Sciences AB, Sweden) density gradient centrifugation. Apoptosis of PBMC was induced by Caesium-137 irradiation with 60 Gray (Gy) and PBMC were resuspended in CellGro serum-free medium (Cell Genix, Freiburg, Germany; 25 × 10^6^ cells/ml). After incubation for 24 h supernatants were dialyzed against ammonium acetate (at a concentration of 50 mM), sterile filtered, frozen and lyophilized. APOSEC from four different donor pigs were pooled for further experiments.

For in vitro experiments PBMC obtained from young healthy volunteers (APOSEC healthy), patients suffering from insulin-dependent diabetes (APOSEC DM), or patients with congestive heart failure NYHA>III (APOSEC CHF) were used (ethics committee vote: EK-Nr 2010/034; 2009/352). Secretome was produced according to the protocol described above; cells were cultured at a concentration of 1 × 10^6^ cells/ml for platelet and a concentration of 2.5 × 10^6^ cells/ml for HUVEC experiments. UltraCulture (Cambrex Corp., North Brunswick, NJ, USA) served as the carrier medium. APOSEC pooled from six to seven donors was used for the respective experiments.

### Porcine closed chest reperfused infarction model and administration of APOSEC

**Fig. 1:**
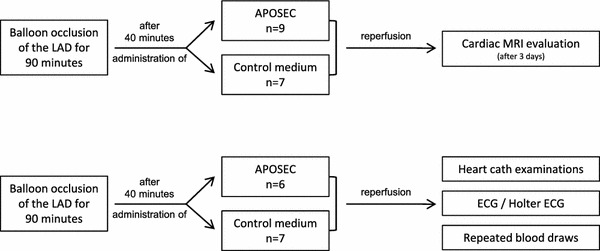
Flow charts of the two experimental settings of the porcine acute myocardial infarction experiments

Animal experiments were approved by the University of Kaposvar, Hungary (vote: 246/002/SOM2006, MAB-28-2005). Two experimental settings were designed (Fig. 1). Pigs (female large whites weighing approximately 30 kg) received 75 mg clopidogrel and 100 mg acetylsalicylic acid as a premedication. At the day of intervention animals were sedated with 12 mg/kg ketamine hydrochloride, 1.0 mg/kg xylazine and 0.04 mg/kg atropine. A Maverick balloon catheter (diameter: 3.0 mm, length: 15 mm; Boston Scientific, Natick, USA) was inserted into the left anterior descending artery (LAD) and inflated after the origin of the second major diagonal branch; ST segment abnormalities were recorded by electrocardiography (ECG). ST-segment resolution was calculated as an ST-segment decrease of >50 % of the initial ST-segment elevation. Additionally, pigs were monitored by Holter ECG during ischemia and until 60 min after reperfusion (Gepa-Med, Vienna, Austria). Forty minutes after the start of the LAD occlusion, the lyophilized secretome from 1 × 10^9^ irradiated apoptotic porcine PBMC or lyophilized serum-free cell culture (resuspended in 250 ml of 0.9 % NaCl solution) was administered intravenously over 25 min. After 90 min occlusion, the balloon was deflated and reperfusion was established. Control coronary angiography was performed to prove the patency of the infarct-related artery and to exclude arterial injury. Euthanasia was performed by the administration of saturated potassium chloride 24 h or 3 days after AMI induction.

### Magnetic resonance imaging

MRI imaging was performed on day three with a 1.5-T clinical scanner (Avanto, Siemens, Erlangen, Germany). Planimetric analysis of MRI images was performed using QMass software (Medis, Leiden, The Netherlands). Similarly to other studies [2], the presence of MVO was evaluated by observing the late hypo-enhancement within a hyper-enhanced region on late enhancement MRI images, 10 min after the administration of intravenous Gadolinium based contrast agent. Previously, infarcted areas were semi-automatically segmented by thresholding the left ventricular myocardium to the mean +2 × SD values of unaffected myocardium. MVO was manually assessed for each subject when areas within the infarcted areas presented low signal intensity (i.e., “dark zones” within “bright” zones). Manual planimetry was used to define the area of MVO for each slice and then areas were multiplied by the slice thickness (8 mm) to get volumetric measurements. Results are given in volume values (cm^3^).

### Bari score analysis

To verify comparable basic conditions between groups prior to balloon occlusion, Bari scores were calculated for all animals based on LAD and CX pre-occlusion angiogram according to the method previously described [49].

### APOSEC content evaluation

APOSEC produced from healthy donors, diabetic patients and CHF patients was evaluated for levels of IL-8, ENA-78, VEGF (all Duoset kits, R&D systems, Minneapolis, USA) following the manufacturer’s instructions. Nitric oxide (NO) was determined by measuring decomposition products nitrite and nitrate with a commercially available colorimetric assay kit (Abcam, Cambridge, UK).

### In vivo platelet function during ischemia/reperfusion

Plasma samples (3.8 % trisodium citrate tubes) were obtained by a venous draw before occlusion (0 h), before balloon deflation (90 min), after reperfusion (240 min) and after 24 h. Secreted platelet activation markers sCD40L, sCD62P, platelet factor-4 (PF-4) and thrombospondin-1 (TSP-1) were measured using commercially available ELISA kits (Uscn, Wuhan, China).

### In vitro platelet function analyses

#### Human platelet isolation

Blood was drawn from eight healthy human volunteers, who declared to be free of any medication for at least 2 weeks. All blood donors gave their informed written consent to the study. They were venipunctered with a 20-G needle and the blood was anticoagulated with one/ten volume of 3.8 % (w/v) trisodium citrate. Immediately after collection, blood was centrifuged at 125 g for 20 min to obtain platelet rich plasma (PRP). To avoid contamination with other cell types only the upper two-thirds of the PRP fraction were used. Platelets were purified by gel filtration using Sepharose 4B columns with HEPES-Tyrode buffer containing 0.5 % human serum albumin as previously described [6]. Experiments with porcine platelets were performed with platelet-rich plasma.

#### Measurement of platelet activation

Isolated platelets were pre-incubated with APOSEC of 2 × 10^5^ cultured cells for 10 min and then stimulated for 5 min with thrombin receptor-activating peptide TRAP-6 (BACHEM, Basel, Switzerland), adenosine diphosphate (ADP; Sigma-Aldrich Corp., St Louis, MO, USA) or collagen (MoeLab, Langenfeld, Germany). Platelets were then either incubated with PE labeled anti-CD62P antibody, FITC-labeled anti-CD63 or FITC-labeled anti-CD40L (Becton–Dickinson, Austria) for 30 min, followed by fixation in 1 % formaldehyde and then analyzed by flow cytometry (FACSCalibur, Becton–Dickinson, Austria).

#### Platelet aggregation experiments

Platelet-rich plasma was stirred in the presence or absence of APOSEC from 2 × 10^5^ cultured cells in an optical 4-channel aggregometer (490-4D, Chronolog Corp., Havertown, PA, USA) at 37 °C for 5 min, thereafter the indicated agonists were added and changes in light transmission recorded over 10 min. After this period, theophylline (300 μM) and adenosine (500 μM) were added to stop further activation. Platelets were centrifuged at 1,000 g for 2 min to obtain supernatant which was analyzed for soluble CD62P, soluble CD40L and thrombospondin (TSP-1) content. ELISA tests for sP-selectin (Quantikine; R&D Systems, Minneapolis, MN, USA) and sCD40L (Bender MedSystems, Vienna, Austria) were performed according to manufacturers’ instructions. Thrombospondin-1 was determined by immunoblotting, as previously described [58].

#### Quantification of intraplatelet VASP phosphorylation

Isolated platelets were incubated with different concentrations of prostaglandin E_1_ (PGE_1_) and APOSEC (2 × 10^5^) for 2 min followed (if indicated) by 5 min of incubation with ADP. Cells were fixed in 1 % formaldehyde for 10 min, permeabilized with 0.5 % triton X-100 and incubated for 45 min with monoclonal antiphospho VASP antibody, clone 22E11 (nanoTools, Teningen, Germany), which detects VASP phosphorylation at serine 239. After a washing step, platelets were incubated with secondary fluorescein isothiocyanate (FITC) conjugated polyclonal anti-mouse IgG antibody (Becton–Dickinson) for 30 min and analyzed by flow cytometry.

### In vivo measurements of vasodilatory mediators during ischemia/reperfusion

Plasma samples (3.8 % trisodium citrate tubes) obtained before LAD occlusion, 90 min after occlusion, after reperfusion and 24 h after AMI induction were evaluated for different vasodilatory mediators. Systemic levels of prostacyclin (PGI_2_) and vasoactive intestinal peptide (VIP) were determined by ELISA technique (Uscn, Wuhan, China; antibodies-online, Aachen, Germany). Nitric oxide was determined as described above.

### In vitro analyses of vasodilatory effects of APOSEC

#### HUVEC culture and immunoblot analysis

Primary human umbilical vein endothelial cells (HUVEC) were obtained from CellSystems (CellSystems Biotechnologie, Troisdorf, Germany) and cultured in endothelial cell growth medium (EGM-2, Lonza, Basel, Switzerland) at 37 °C. For Western Blot analysis, 3 × 10^5^ cells were seeded in six-well plates and cultured in EGM-2 medium. 24 h later, cells were incubated with lyophilized APOSEC from 2.5 × 10^6^ cells or lyophilized control medium, resolved in EGM-2 medium without growth factors, for 60 min (phospho-eNOS detection) or 24 h (iNOS detection). Western blot analysis was performed as described previously [45]. Briefly, HUVEC were lysed in SDS-PAGE loading buffer, sonicated, centrifuged, and denatured before loading. SDS-PAGE was conducted on 8–18 % gradient gels (GE Healthcare, Uppsala, Sweden). The proteins were then electro-transferred onto nitrocellulose membranes (Bio-Rad, Hercules, CA, USA). Immunodetection was performed with either a rabbit polyclonal anti-inducible nitric oxide synthase (iNOS) antibody (Cell Signaling Technology, Inc., Danvers, MA, USA), phospho e-NOS antibody (Cell Signaling Technology, Inc.) or a mouse monoclonal anti-GAPDH antibody (Acris, Herford, Germany) followed by horseradish peroxidase-conjugated goat anti-rabbit or goat anti-mouse IgG antisera (both 1:10,000; GE Healthcare). Reaction products were detected by chemiluminescence with the ChemiGlow reagent (Biozyme Laboratories Limited, South Wales, UK) according to the manufacturer’s instructions.

#### Coronary perfusion assay

Coronary perfusion assay was performed as described previously [1]. Hearts were obtained from untreated, sacrificed domestic pigs and transferred to the laboratory in a modified Krebs-Henseleit buffer solution. Coronary arteries were dissected from the heart and cut in 4 mm thick rings. Each coronary segment was mounted in a temperature-controlled 10 mL tissue bath containing a modified Krebs-Henseleit buffer solution. To measure circular wall tension, the rings were suspended between two L-shaped pins in a myograph. After approximately 1 hour, vessels were contracted with endothelin-1 (30 nM; Calbiochem, Darmstadt, Germany). APOSEC was added to the probes in different concentrations (dose escalation) and changes in arterial wall tension were measured. In some experiments NOS inhibitor L-NG-Nitro arginine methyl ester (L-NAME) was added.

#### Immunohistochemical evaluation of coronary rings

Coronary rings isolated according to the above described procedure were incubated for 60 min in the presence or absence of APOSEC. Rings were fixed in 10 % neutrally buffered formaldehyde solution and embedded in paraffin. The tissue samples were stained with hematoxylin–eosin (HE). For immunohistochemical stainings an antibody recognizing eNOS, phosphorylated at Ser 1,177 (Biorbyt, Cambridge, UK) was applied. Tissue samples were evaluated on an Olympus AX70 microscope (Olympus Optical Co. Ltd., Tokyo, Japan) and captured digitally using Meta Morph v4.5 software (Molecular Devices, Sunnyvale, USA).

### Statistical analysis

Results are depicted as mean ± standard error of the mean and were analyzed by student’s *t* test or repeated measures analysis of variance (ANOVA) followed by Bonferroni correction. Data analysis was performed with SPSS 18.0 (SPSS inc., United States). A *p* value less than 0.05 was regarded as statistically significant (asterisk indicates *p* < 0.05; double asterisk indicates *p* < 0.01).

## Results

### APOSEC reduces MVO in a porcine AMI model

APOSEC has recently been shown to effectively reduce myocardial damage during AMI [39]. To define the impact of APOSEC on MVO, pigs were evaluated 3 days after myocardial infarction by MRI. Areas of MVO were significantly lower in pigs treated with APOSEC when compared to control animals (Table 1; APOSEC: 0.3 ± 0.1 cm^3^; control: 0.8 ± 0.1 cm^3^; *p* = 0.04). This finding was confirmed by cardiac catheterization, as the corrected thrombolysis in myocardial infarction (TIMI) frame count was significantly lower in APOSEC treated animals (28.7 ± 1.9 vs. 44.4 ± 3.6; Table 2). In addition, the myocardial blush grade, which directly reflects myocardial tissue perfusion, was significantly better in APOSEC treated pigs (mean grade 1.3 ± 0.3 vs. 2.5 ± 0.3; Table 2).

**Table 1:** MVO analysis

	Group	MVO (cm^3^)	Qualitative
1	APOSEC	0	Not visible
2	APOSEC	0.427	Small
3	APOSEC	0	Not visible
4	APOSEC	1.24	Small
5	APOSEC	0	Not visible
6	APOSEC	0.56	Small
7	APOSEC	0	Not visible
8	APOSEC	0	Not visible
9	APOSEC	0.91	Small
10	Control	0.86	Small
11	Control	0.76	Small
12	Control	1.04	Small
13	Control	0.26	Very small
14	Control	0.96	Small
15	Control	0.72	Small
16	Control	0.97	Small

Pigs were evaluated 3 days after induction of AMI for areas of MVO

APOSEC treated animals had significant smaller areas of impaired microvascular perfusion when compared to control animals (APOSEC: 0.3 ± 0.1; control: 0.8 ± 0.1; *p* = 0.04)

**Table 2:** Cardiac catheterization analysis

	Control	APOSEC	*p* value
Corr. TMI frame count	44.4 ± 3.6	28.7 ± 1.9	0.022
Myocardial blush grade	1.3 ± 0.3	2.5 ± 0.3	0.033

Corrected TIMI frame counts were lower in animals treated with APOSEC indicating a good microvasculature perfusion (*p* = 0.022)

Additionally, animals from the APOSEC group had a significantly higher myocardial blush grade than control pigs (*p* = 0.033)

*n* = 6–7

### BARI scores

To rule out the possibility that differences in MVO could be a result of differences in the coronary vascularisation, Bari scores were determined from pre-interventional angiographies. A homogenous distribution of coronary vessels was found between groups (Suppl. Fig. 1a).

### Area at risk measured by MRI

During the early phase of AMI, the area at risk (AAR) determines the zone of ischemic injured myocardium. To confirm that ischemic areas were comparable in both groups we analyzed T2-weighted images in the MRI analysis 3 days after LAD occlusion. No differences in the AAR could be found between the two groups (control: 22.9 ± 2.2 vs. APOSEC: 20.2 ± 1.4; *p* = 0.294; Suppl. Fig. 1b), evidencing that the size of hypoperfused myocardium at the time of the ischemic episode was similar in the groups.

### Haemodynamic monitoring and ECG data

Haemodynamic monitoring showed a trend towards better left ventricle contraction capacity (dP/dt/P) in APOSEC group, as compared to control group (27.2 ± 20.6 vs. 17.4 ± 4.0 min^−1^). On-line ECG monitoring during coronary occlusion and reperfusion showed ST segment resolution in four out of six animals in the APOSEC group compared to only one out of seven pigs in the medium group. Holter ECG evaluations revealed a reduction of ventricular arrhythmias (expressed in total number of extrasystoles, couplet, triplet, and ventricular tachycardias) during coronary occlusion and the perfusion period (Table 3).

**Table 3:** Rhythmological evaluation

	ST‐resolution		VES		Couplet		Triplet		VT	
	Control	APOSEC	Control	APOSEC	Control	APOSEC	Control	APOSEC	Control	APOSEC
During occlusion	–	–	238.7 ± 161.5	28.0 ± 11.0	10.7 ± 7.1	4.6 ± 3.0	10.7 ± 8.5	0.2 ± 0.2	5.7 ± 3.4	2.4 ± 1.9
After reperfusion	1/7	4/6	92.3 ± 31.0	49.0 ± 35.8	18.8 ± 8.6	8.0 ± 5.6	4.8 ± 3.3	3.4 ± 2.0	3.2 ± 1.9	3.4 ± 2.4

ECG and Holter-ECG analyses revealed lower rates of persisting ST abnormalities and significantly fewer episodes of arrhythmias in pigs receiving APOSEC

This was shown for *VES* ventricular extrasystole, coulets, triplets, *VT* ventricular tachycardia

*n* = 6–7

### APOSEC inhibits platelet aggregation in vivo and in vitro

**Fig. 2:**
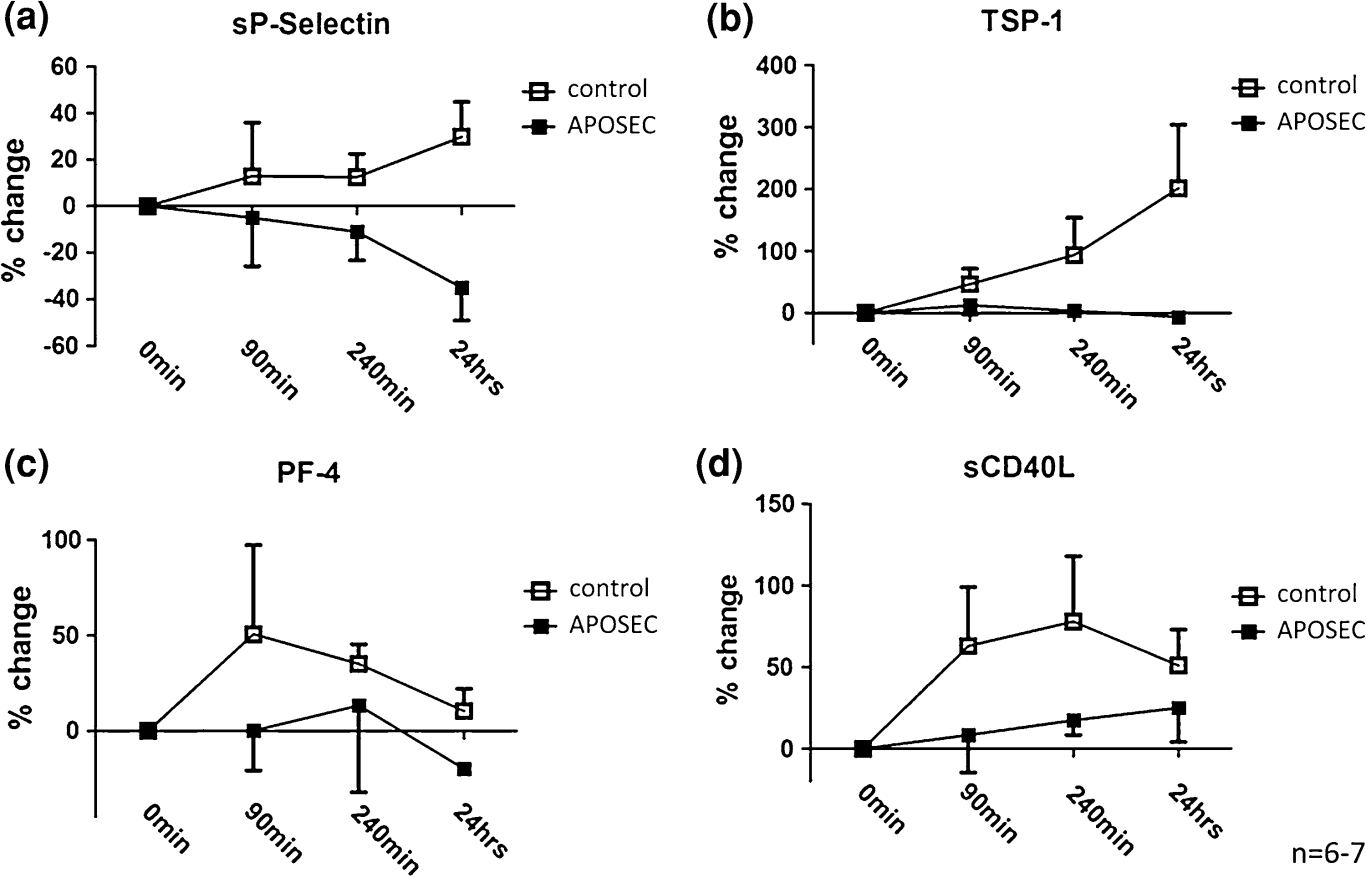
Platelet activation markers. Soluble activation markers (sP-selectin, TSP-1, PF-4 and sCD40L) were reduced in APOSEC treated animals when compared to control pigs (**a**–**d**)

**Fig. 3:**
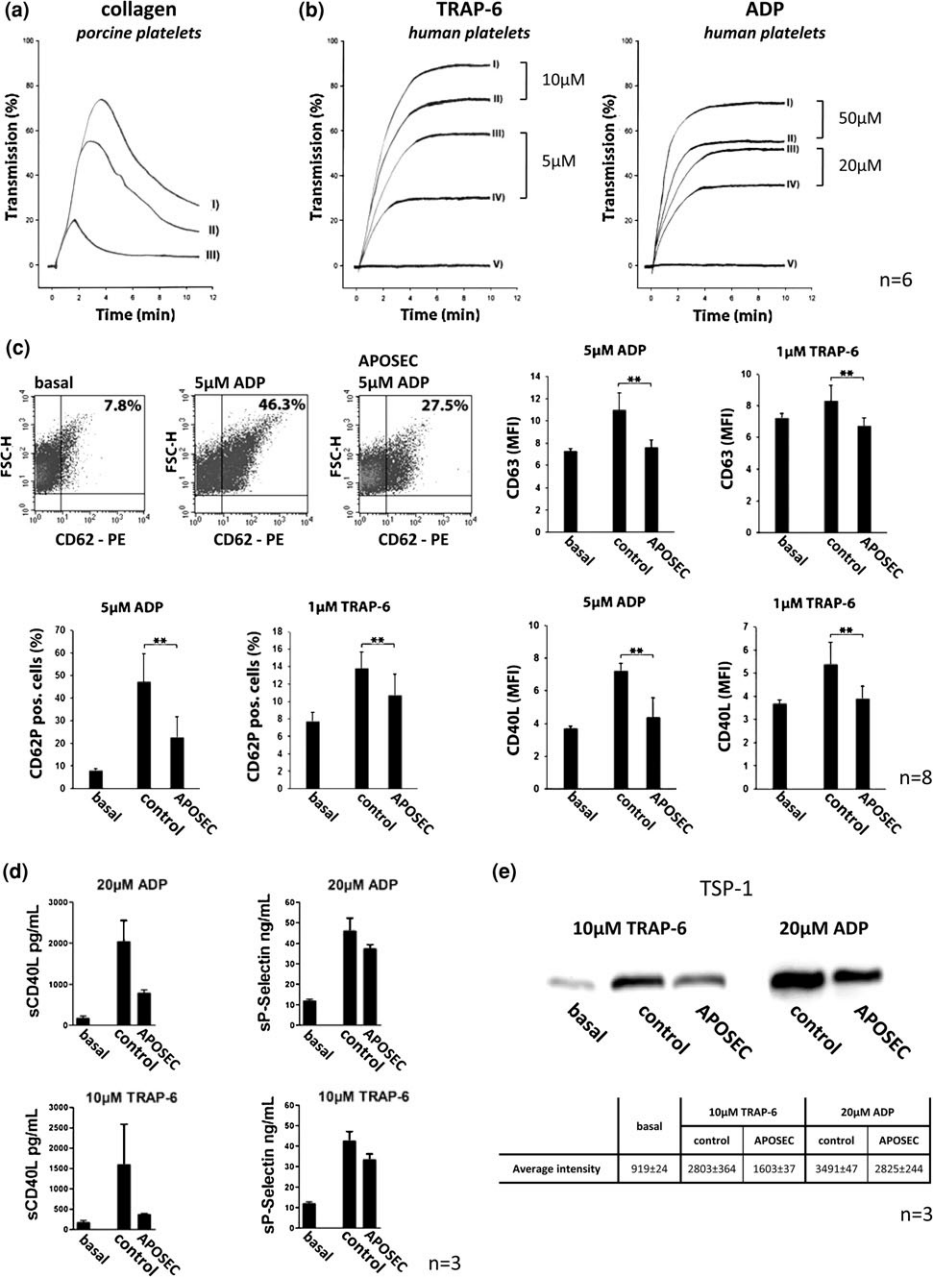
Influence of APOSEC on platelet aggregation and activation. Aggregation experiments are depicted in **a**: **I** collagen (10 μg/mL); **II** collagen (10 μg/mL) + APOSEC 1 × 10^6^; **III** collagen (10 μg/mL) + APOSEC 1 × 10^7^, **b**: *left*: **I** TRAP-6 (10 μM); **II** TRAP-6 (10 μM) + APOSEC 2 × 10^5^; **III** TRAP-6 (5 μM); **IV** TRAP-6 (5 μM) + APOSEC 2 × 10^5^; **V** basal/APOSEC 2 × 10^5^, *right*: **I** ADP (50 μM); **II** ADP (50 μM) + APOSEC 2 × 10^5^; **III** ADP (20 μM); **IV** ADP (20 μM) + APOSEC 2 × 10^5^; **V** basal/APOSEC 2 × 10^5^, **c**: surface exposure of CD62P, CD63, and CD40L after stimulation with ADP or TRAP-6 in the presence or absence of APOSEC. Influence of APOSEC on secreted activation markers is shown in **d** and **e**. Levels of sCD40L, sP-selectin, and thrombospondin-1 were lower in the supernatant of APOSEC treated platelets when compared to controls

Since platelets are the major contributor to MVO we hypothesized that APOSEC has a direct influence on platelet function. Systemic platelet activation markers in plasma, obtained at different time points after AMI induction, were measured. Levels of sP-selectin, TSP-1, PF-4 and sCD40L were lower in the APOSEC group when compared to control animals (Fig. 2a–d). These in vivo findings were confirmed by in vitro experiments. Isolated porcine and human platelets were stimulated with different concentrations of collagen, ADP and TRAP-6 with or without pre-incubation of APOSEC. As measured by light transmittance aggregometry, platelet aggregation could be inhibited by the addition of APOSEC (Fig. 3a, b), both in a maximal and a half-maximal stimulation model.

The inhibitory effect of APOSEC on platelets was further characterized by measuring surface expression of different platelet activation markers. Levels of CD62P, CD63 and CD40L were significantly decreased after treating platelets with APOSEC, indicating an inhibitory role of APOSEC during platelet activation (Fig. 3c). These findings were corroborated by the evaluation of secreted activation factors in the supernatant of aggregation experiments. As determined by ELISA, concentrations of sCD40L and sCD62P were significantly lower after treating platelets with APOSEC (Fig. 3d). Thrombospondin has recently been described as a sensitive and stable parameter to monitor in vitro platelet activation. We therefore evaluated supernatants for amounts of secreted TSP-1 isoforms by western blots. There was a strong band of 140 kD TSP-1 detectable after ADP and TRAP-6 activation, which was reduced upon coincubation of platelets with APOSEC (Fig. 3e).

### Enhanced VASP phosphorylation by APOSEC

**Fig. 4:**
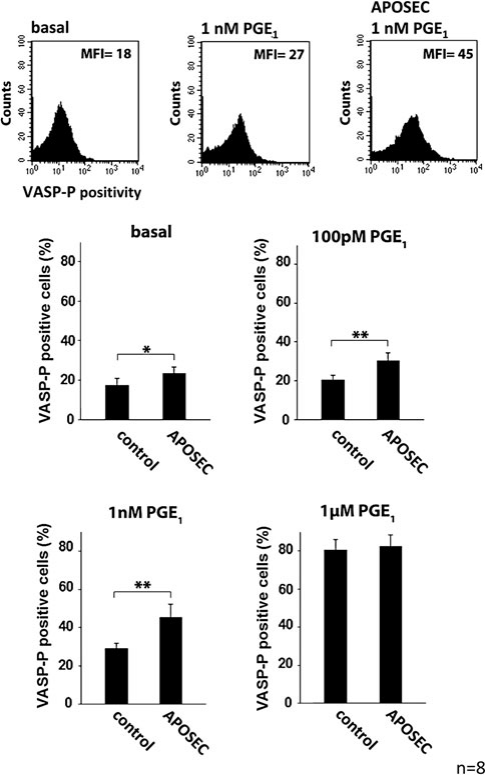
In vitro effect of APOSEC on VASP-phosphorylation. Platelets from healthy donors in the presence and absence of APOSEC were analyzed for basal and PGE_1_ induced VASP-phosphorylation. Co-incubation with APOSEC led to significantly increased levels of intraplatelet phosphorylated VASP

VASP in its phosphorylated form represents a negative regulator of platelet activation. We could show that incubation of isolated human platelets with APOSEC led to an increase of intraplatelet phosphorylated VASP. In addition, coincubation of platelets with both APOSEC and different submaximal effective concentrations of PGE_1_ increased VASP-phosphorylation in a synergistic way (Fig. 4). This is of special interest as PGE_1_ represents a physiological relevant inhibitor of platelet function that acts through an increase of intraplatelet cAMP.

### APOSEC induces coronary vasodilation

**Fig. 5:**
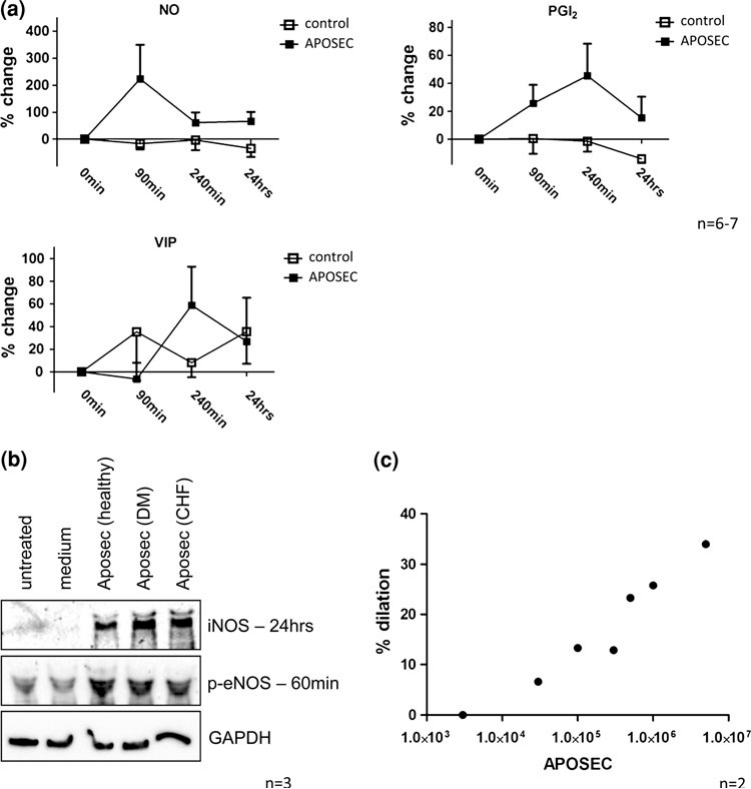
Impact of APOSEC on vasodilation. Vasodilatory mediators (NO, PGI_2_, VIP) were increased in AMI treated animals when compared to control pigs (**a**). HUVEC treated with APOSEC evidenced a strong induction of iNOS and p-eNOS (**b**, one representative experiment; *n* = 3). Myographic analysis of isolated coronary vessel rings showed a direct dose-dependent effect of APOSEC on vascular tonus (**c**)

The role of vasodilators in the prevention and treatment of MVO is well described [33]. We therefore assessed vasodilatory mediators in serum samples obtained from the AMI animal model. Both, NO and PGI_2_ were found to be increased after administration of APOSEC when compared to control animals (Fig. 5a). In addition, HUVEC upregulated iNOS expression after 24 h of co-incubation with APOSEC as determined by western blot. eNOS expression was not altered (data not shown), however, the active phosphorylated form of eNOS was increased 60 min after treating HUVEC with the compound (Fig. 5b). p-eNOS expression was also found elevated in coronary rings 60 min after treatment with APOSEC as determined by immunohistochemical stainings (Suppl. Fig. 4).

Finally, we evaluated direct vasodilatory effects of APOSEC. In APOSEC preparations NO was found in significant concentrations (12 × 10^6^ mL: 39.5 nM; 1.2 × 10^6^ mL: 16.9 nM: 0.12 × 10^6^ mL: 1.3 nM), however, PGI_2_ or VIP was not detectable. Myographic evaluations using isolated coronary arterial segments corroborated this finding. Treating coronary rings with APOSEC resulted in a significant dilation of the vessels in a dose dependent manner (Fig. 5c). This effect was not related to a de novo production of NO, since blocking NO synthesis with L-NAME had no effect on vasotonus (Suppl. Fig. 2).

### APOSEC from healthy donors, diabetic patients and CHF patients show comparable properties

To address the question if the observed effects of APOSEC are limited to healthy donors, we produced APOSEC from diabetic patients and patients suffering from CHF. Levels of three reference cytokines (IL-8, ENA-78, VEGF), which are known to be highly abundant in APOSEC [38], and NO were determined. No differences between APOSEC (healthy), APOSEC (DM) and APOSEC (CHF) were observed (Suppl. Fig. 3a). The functional relevance of this finding was further evaluated in platelet aggregation and vasodilation experiments. APOSEC (DM) and APOSEC (CHF) were similar effective in inhibiting platelet function (Suppl. Fig. 3b) and in inducing p-eNOS and iNOS expression in HUVEC (Fig. 5b) when compared to APOSEC (healthy).

## Discussion

This study gives first evidence that APOSEC effectively reduces MVO in a clinically relevant ischemia/reperfusion AMI model. This finding was associated with an improvement in the myocardial blush grade and corrected TIMI frame count, two clinically established parameters of microvascular patency. Moreover, resolution of ECG alterations during experimental occlusion and reperfusion were mediated by treating animals with APOSEC. The impact of APOSEC on two major contributors of MVO was tested in vitro. Co-incubation of platelets and APOSEC led to an increase of phosphorylated VASP, consecutively inhibiting platelet aggregation in vitro. Treating HUVEC with APOSEC resulted in an induction of iNOS and p-eNOS. Additionally, direct vasodilatory effects of APOSEC were shown in myographical evaluations of isolated coronary arterial rings.

For a long time, beneficial effects in stem cell therapy were contributed solely to cellular mediated mechanism. Recently, this concept was challenged by works showing that paracrine signalling may be a significant additional mode of action [19, 41, 52, 53]. The importance of releasing pro-angiogenic and cytoprotective factors during AMI has already been shown for mesenchymal as well as for bone marrow derived stem cells [3, 14, 36]. We have recently expanded the concept of regeneratory, paracrine factors derived from stem cell, by showing that the secretome of apoptotic PBMC attenuates myocardial infarction [39]. The major advantage of PBMC over stem cells is that they are a lot easier to access. Although secretome of stem cells and PBMC both mediate similar effects, their secreted factors slightly differ. In a protein chip array study Wollert and colleagues showed that out of 174 secreted factors, 25 factors were present in higher concentrations in bone marrow supernatants, and ten factors were found in higher concentrations in peripheral blood leucocytes [36]. To the best of our knowledge, our group was first to utilize the potential of paracrine factors derived from PBMC in an experimental AMI setting. Consequently, we have addressed features of APOSEC relevant for microvascular obstruction in this subsequent study.

After re-establishing blood flow in the occluded epicardial vessel, the integrity of the microcirculation in the vicinity of the post-ischemic myocardium is pivotal for a patient’s prognosis. An open microvasculature was shown to supply infarct related myocardium with blood and avoiding myocyte necrosis [10, 42]. It is of utmost importance to maintain this residual blood flow within the AAR, since there is sufficient in vitro and in vivo evidence of viable myocardium hours to days after coronary occlusion [32, 44, 63]. If the preservation of microvascular flow fails, viable myocardium is gradually lost. It is a currently accepted notion that platelets are causative for microvascular dysfunction by releasing vasoconstrictive substances [21], by forming microemboli [28, 57] or by intravasal thrombus formation in the microcirculation [7]. Moreover, experimental evidence indicates that the detrimental effect of platelets is dependent on their activation status [64]. Relevant to MVO are the observations of Barrabes et al. [8], who showed in a porcine AMI model that ischemic injury triggers macro- and microvascular platelet deposition even in distant areas not related to the occluded coronary artery. This leads to impairment in coronary flow reserve and contractile function. With advances in understanding the pathophysiology of microvascular malperfusion, different therapeutic strategies inhibiting platelet function have been proposed. However, to date only the application of monoclonal antibodies blocking GPIIb–IIIa receptor improved microvascular flow and subsequently reduced infarct size in animal models [37]. This effect could be confirmed in double-blind randomized trials which have led to a class IIA recommendation of use of anti GP IIb/IIIa in the ACC/AHA guidelines [5, 46].

Currently there is no standard in measuring microvasculature dysfunction in vivo. Several techniques including coronary angiography, contrast echocardiography, and MRI are used clinically and experimentally to describe MVO. Each of these techniques measures slightly different biological and functional parameters [9]. We therefore decided to confirm our MRI data with cardiac catheterization measurements. A low TIMI frame count indicates a sufficient blood flow in the small vessels; on the other hand a high TIMI frame count is associated with microvascular occlusion [18]. The angiographic myocardial blush grade is a standard method to clinically assess myocardial tissue perfusion [24]. It has a direct impact on patients’ prognosis since a persistently abnormal myocardial blush grade was shown to result in reduced functional parameters in the long-term [30].

No-reflow phenomenon is known to be associated with persistent ST-elevation and ventricular arrhythmias [12, 31]. About 25 % of patients ST-segment abnormalities persist even though coronary blood flow has been restored. Therefore, we sought to determine whether APOSEC has an effect on ECG alterations during AMI. As shown in Table 3, infusion of APOSEC led to a normalization of ST segment alterations in the majority of treated animals. In addition, arrhythmic episodes were lower in the APOSEC group during occlusion and reperfusion.

The beneficial effects of APOSEC on MVO in our porcine in vivo AMI model are in line with in vitro data obtained after exposure of porcine platelets to APOSEC. Co-incubation of platelets with APOSEC prevented platelet aggregation triggered by collagen. Based on these findings further experiments were performed with human platelets and similar effects could be observed. The addition of TRAP-6 at a final concentration of 10 μM and ADP at a concentration of 50 μM caused platelets to fully aggregate and this aggregation was effectively impaired by preincubation of platelets with APOSEC. Interestingly, APOSEC derived from PBMCs isolated from diabetic and heart insufficiency patients triggered the same effects compared to APOSEC obtained from healthy patients.

Platelet surface P-selectin is considered to be the “gold standard” marker of platelet activation and was significantly reduced after preincubation of purified platelets with APOSEC [43]. This finding was further supported by reduced platelet surface markers CD63 and CD40L and lower concentrations of sCD40L, sP-selectin, and TSP-1 in the supernatant of APOSEC exposed platelets.

A recent paper by Köhler et al. [35] has provided profound evidence that the phosphorylation state of VASP is crucially important for the extent of myocardial ischemia/reperfusion injury. Increased intra-platelet phosphorylated VASP was shown to prevent platelet activation and platelet-neutrophil complex formation during AMI. These findings were meticulously confirmed with VASP knock-out animals, bone marrow chimeric animals and a platelet transfer model. Therapeutic augmentation of phosphorylated VASP using a guanylyl cyclase activator was shown to be effective in a rodent animal model [54]. We consequently addressed the question whether APOSEC is capable to induce VASP phosphorylation in platelets. Indeed, we were able to show that APOSEC led to an increase of phosphorylated VASP, and these effects of APOSEC could be observed in the absence as well as in the presence of submaximal effective concentrations of prostaglandin E_1_.

Besides platelet activation and aggregation, endothelial dysfunction in the small coronary vasculature is another major component in the pathophysiology of the no-reflow phenomenon. During reperfusion the endothelium is injured by oxygen free radicals resulting in an impaired endothelium-dependent vasodilation [55]. Besides, aspirates from coronary arteries obtained during PCI were shown to contain vasoconstrictor factors [34]. The concept of increased vasomotor tone in the area of MVO is supported by several clinical trials, testing different vasodilators during PCI. Currently, adenosine, verapamil or nitroprusside are a recommended therapeutic option for the treatment of no-reflow [25, 33]. Since “classical” vasodilatory drugs have been proven beneficial in the setting of no-reflow, we investigated whether APOSEC has also an effect on the vasomotor tone. In plasma samples obtained after AMI induction, systemic levels of vasodilatory mediators were heightened. In line with this finding we were able to show that HUVEC upregulated iNOS and p-eNOS expression after application of APOSEC. Besides these long-term effects on NO synthases, also a direct vasodilatory impact of APOSEC on isolated coronary vessel rings was observed, which was independent of NOS activity (Fig. 5, Suppl. Fig. 2).

Despite the effects of APOSEC on expression and activation of nitric oxide synthases, some immediately occurring effects might (also) be caused directly by biologically active compounds residing in APOSEC. In this regard, the identification of significant amounts of nitrite/nitrate in APOSEC preparations might be of central importance. As APOSEC is extensively dialyzed we can exclude the possibility that NO decomposition products nitrite and nitrate are present in APOSEC. Therefore, we consider it safe to conclude that protein adducts of nitric oxide represent a biologically active ingredient of APOSEC. The NO-axis has been shown to mediate cardioprotective signalling [27, 29] and locally liberated NO might be responsible for some of the immediate effects of APOSEC, especially those we could observe in experiments dealing with vascular tension and platelet activation. Specifically, such a mechanism would be in line with APOSEC-mediated vasodilation that occurs in L-NAME treated coronary rings and the finding that APOSEC enhances VASP phosphorylation even when applied alone (i.e., in the absence of prostaglandin E_1_).

For the current study APOSEC was produced and tested in allogeneic fashion, hence all experiments were performed with APOSEC obtained from (genetically non-identical) donors of the same species. In order to extend to the clinical reality we also obtained APOSEC from diabetic and heart failure patients. As shown in Suppl. Figs. 3 and 5b concentrations of reference cytokines and results of functional assays (platelet aggregation, iNOS, p-eNOS induction in HUVEC) were comparable in APOSEC derived from healthy and diseased patients. Consequently, these data suggest autologous (“autotransplantation” of APOSEC derived from a diseased patient) as well as allogeneic source (APOSEC derived from healthy donors, similar to plasma derivatives) might be feasible options for patients suffering of hypoxia induced ischemic conditions. In respect to planned autologous and allogeneic APOSEC production strict regulatory prerequisites (e.g., virus inactivation, potency assays, and mandated GMP facilities) have to be met in order to reach human clinical trials.

## Conclusion and outlook

APOSEC is a compound made of soluble factors secreted by irradiated PBMC and has previously been shown to abrogate myocardial damage in a large animal ischemia reperfusion AMI model. We have evidenced that this “biological” induces peri-infarct conditioning and homing of autologous c-kit positive cells into the hypoxic myocardium and thus prevents ventricular remodeling [39]. These data are in line with other published reports showing that secretomes derived from hematopoietic and mesenchymal stem cells display similar features in experimental ischemic conditions [13, 20, 36, 56].

In this study we present in vivo and in vitro data showing that APOSEC is able to abrogate platelet aggregation, induce vasodilation and attenuates microvascular obstruction in an experimental large animal AMI model. We believe that APOSEC combines the following favorable features (1) obtaining PBMC for APOSEC production is simple compared to stem cell based compounds; (2) minimal or no antigenicity owing to protein-only content; (3) potentially “off the shelf” utilization in the setting of AMI. Our data on microvascular obstruction adds further support to the notion that APOSEC initiates a multiplicity of favorable (pleiotropic) effects in hypoxic conditions.

The “stem cell centric vision” (e.g., Bolli et al. [11]) in regenerative medicine is currently under critical appraisal [61] and our study underlines the credibility of the “paracrine hypothesis” [19, 36]. Good clinical manufacturing of PBMC derived APOSEC, which is currently in the planning phase, will pave the way to first human trials.

### Limitation

In all in vitro and in vivo experimental settings (porcine reperfused AMI model, in vitro experiments using porcine and human cells), APOSEC was tested in a syngeneic fashion only, in order to obviate inter-species influences.

## Electronic supplementary material

Below is the link to the electronic supplementary material.

**Suppl. Figure 1** Bari sore analysis of cardiac catheterization films before the intervention showed no difference between groups indicating an even distribution of coronary collateralization (a). Area at risk determined on day 3 by MRI was comparable in both groups (b). **Suppl. Figure 2** Impact of NOS inhibition on coronary ring assays. The addition of L-NAME had no effect on the direct vasodilatory capacity of APOSEC. **Suppl. Figure 3** Comparison of APOSEC from healthy and diseased donors. There was no difference between APOSEC from healthy donors, APOSEC from diabetic patients and APOSEC from CHF patients regarding reference cytokines and NO (a). Additionally, APOSEC from the three donor groups evidenced similar anti-aggregatory features as determined by aggregometry (b). I) collagen (10 μg/mL) II) collagen (10 μg/mL) + APOSEC (healthy) 2.5*10^6^ III) collagen (10 μg/mL) + APOSEC (DM) 2.5*10^6^ IV) collagen (10 μg/mL) + APOSEC (DM) 2.5*10^6^. **Suppl. Figure 4** p-eNOS expression of coronary ring assays. Incubation of coronary rings with APOSEC resulted in an increase of intracellular p-eNOS when compared to control experiments. (PDF 592 kb)
