# Supplementary figures and images for: Secretome of apoptotic peripheral blood cells (APOSEC) attenuates microvascular obstruction in a porcine closed chest reperfused acute myocardial infarction model: role of platelet aggregation and vasodilation
Source: Basic Res Cardiol. 2012 Aug 17;107(5):292. doi: 10.1007/s00395-012-0292-2 (PMC3442164; doi:10.1007/s00395-012-0292-2)

# Suppl. Figure 1

(a)

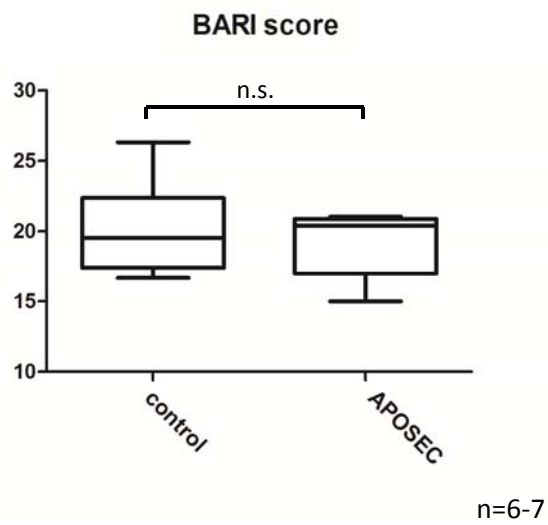

(b)

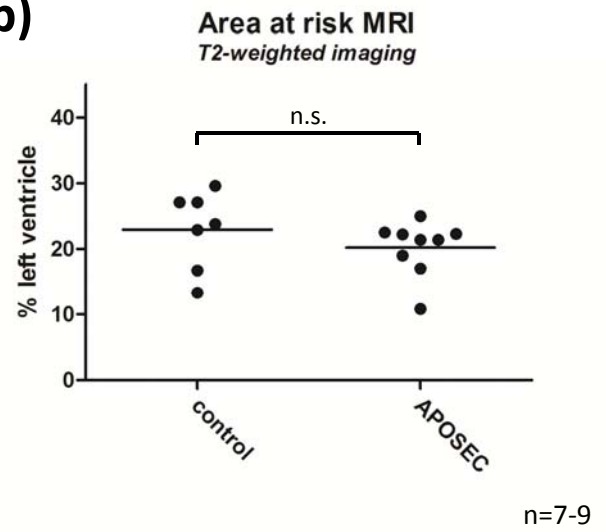

## Suppl. Figure 2

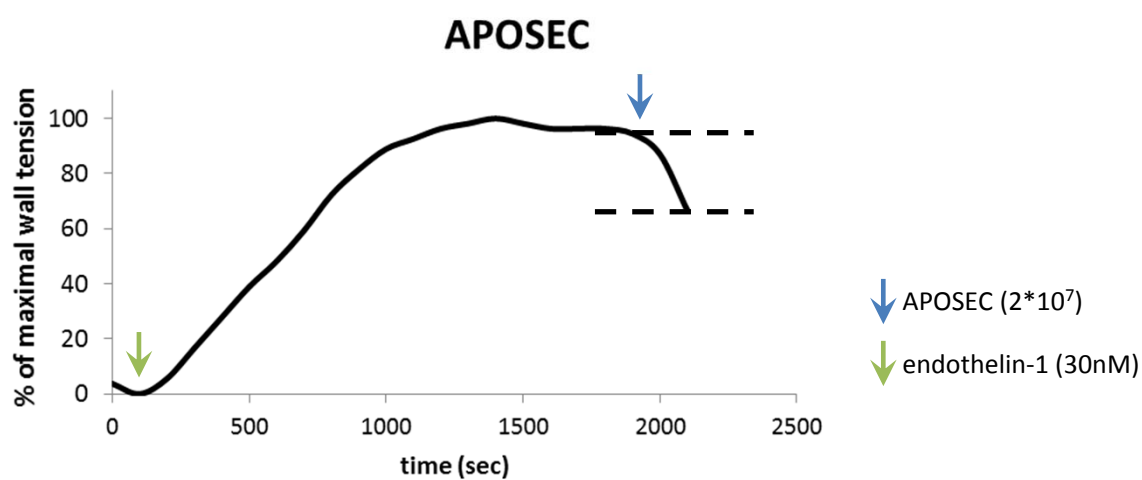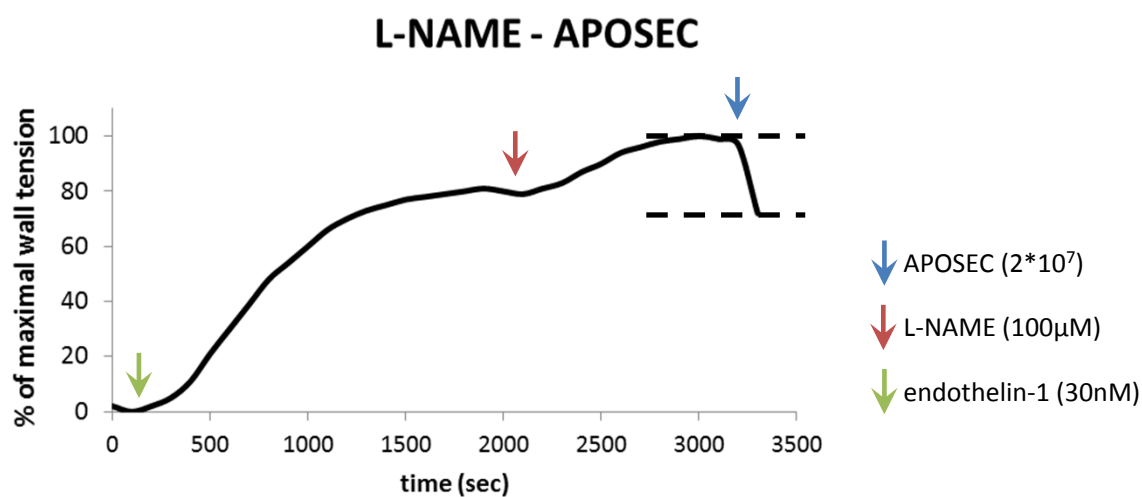

# Suppl. Figure 3

(a)

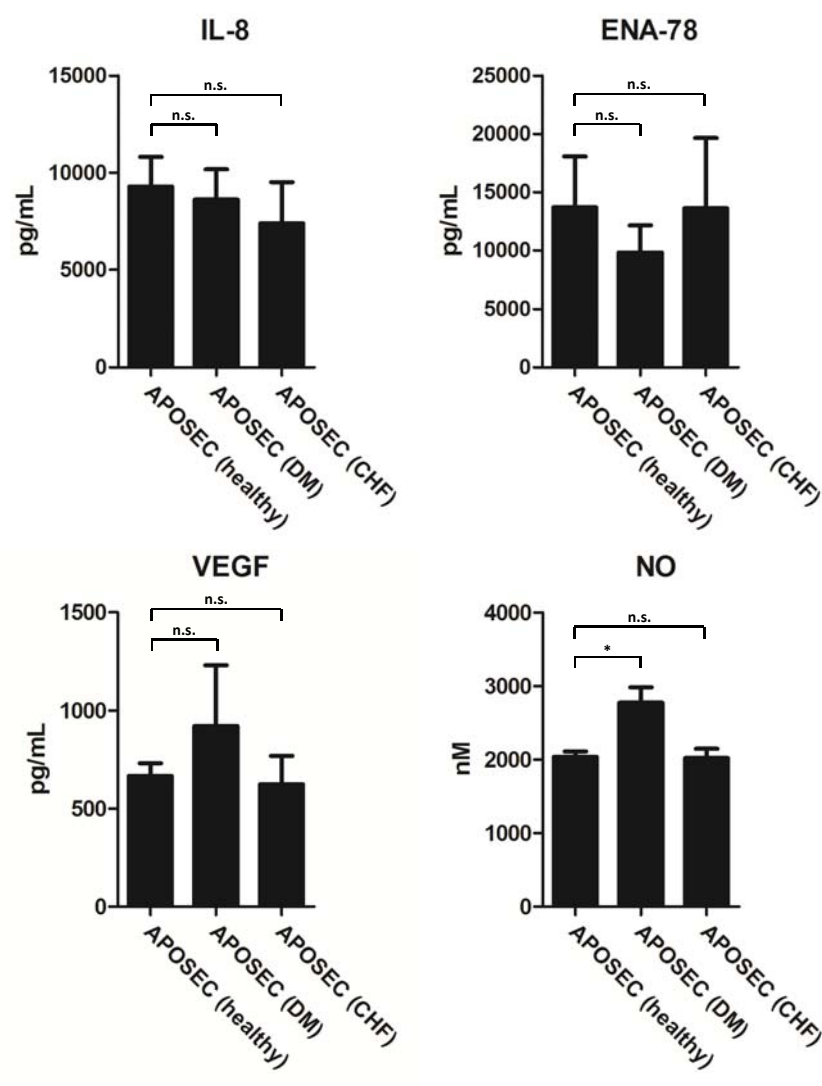

(b)

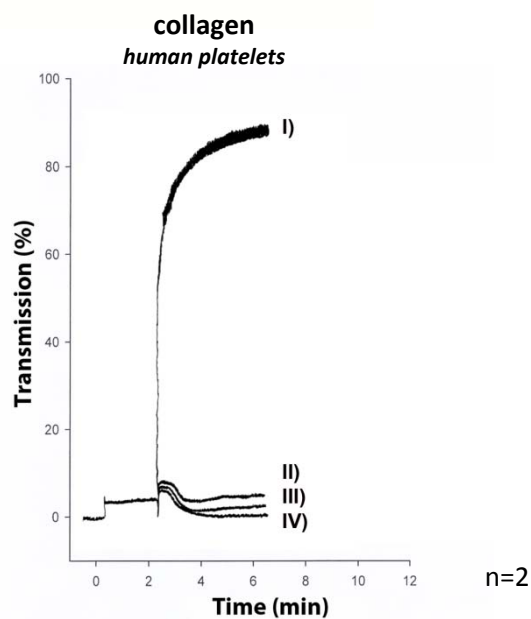

# Suppl. Figure 4

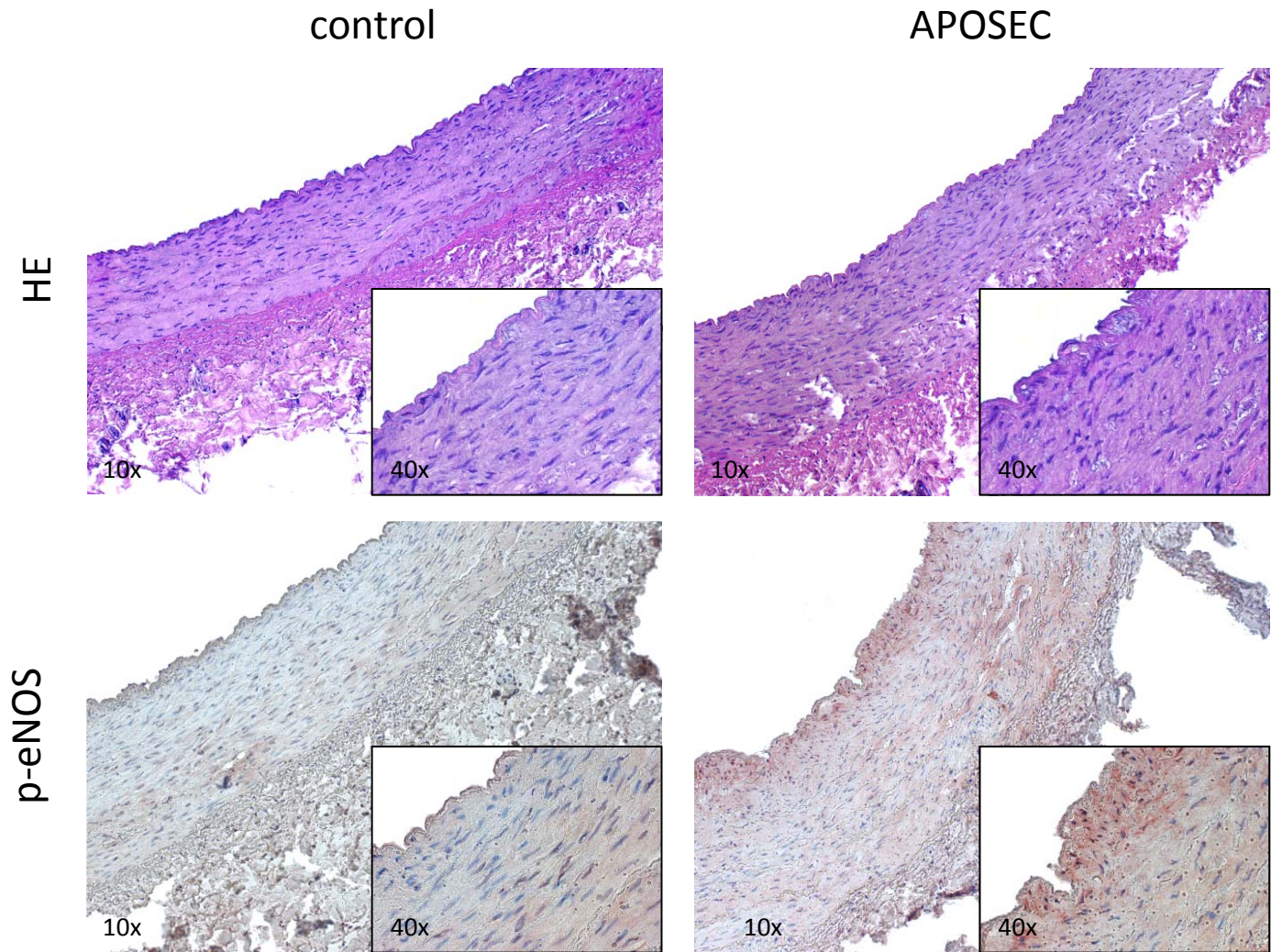

n=3

Supplement: Supplementary file 1 — Suppl. Figure 1 Bari sore analysis of cardiac catheterization films before the intervention showed no difference between groups indicating an even distribution of coronary collateralization (a). Area at risk determined on day 3 by MRI was comparable in both groups (b). Suppl. Figure 2 Impact of NOS inhibition on coronary ring assays. The addition of L-NAME had no effect on the direct vasodilatory capacity of APOSEC. Suppl. Figure 3 Comparison of APOSEC from healthy and diseased donors. There was no difference between APOSEC from healthy donors, APOSEC from diabetic patients and APOSEC from CHF patients regarding reference cytokines and NO (a). Additionally, APOSEC from the three donor groups evidenced similar anti-aggregatory features as determined by aggregometry (b). I) collagen (10 μg/mL) II) collagen (10 μg/mL) + APOSEC (healthy) 2.5*106 III) collagen (10 μg/mL) + APOSEC (DM) 2.5*106 IV) collagen (10 μg/mL) + APOSEC (DM) 2.5*106. Suppl. Figure 4 p-eNOS expression of coronary ring assays. Incubation of coronary rings with APOSEC resulted in an increase of intracellular p-eNOS when compared to control experiments. (PDF 592 kb) [file 395_2012_292_MOESM1_ESM.pdf]
